# P008. Alexithymia and chronic migraine with medication overuse: what relationship?

**DOI:** 10.1186/1129-2377-16-S1-A150

**Published:** 2015-09-28

**Authors:** Sara Bottiroli, Federica Galli, Michele Viana, Grazia Sances, Marta Allena, Natascia Ghiotto, Elena Guaschino, Giorgio Sandrini, Cristina Tassorelli, Giuseppe Nappi

**Affiliations:** Headache Science Centre (HSC), C. Mondino National Neurological Institute, Pavia, Italy; Department of Health Sciences, University of Milan, Milan, Italy; Department of Brain and Behavioural Sciences, University of Pavia, Pavia, Italy; University Consortium for Adaptive Disorders and Head pain, Pavia, Italy

## Background

Alexithymia is a personality trait characterized by the inability to identify and express emotions. Neuroimaging studies showed specific neural correlates in alexithymic subjects [1] and pathological scores of alexithymia in several chronic pain populations and in episodic migraine [2, 3]. There is also evidence of a positive association between alexithymia, depression, and anxiety in migraine patients. So far, no study has evaluated alexithymia in medication-overuse headache patients (MOH) (progressed by migraine) versus episodic migraine patients (MIG). The present study was aimed to evaluate whether MOH individuals differ from MIG as regards alexithymia scores and to investigate the association of alexithymia with headache characteristics.

## Materials and methods

We recruited 99 patients suffering from MOH (n=54; 81.5% female; age: 41.6±10.9) evolved from migraine (chronic migraine + MOH) or MIG (n=45; 71.6% female; age: 41.0±9.3) at the Headache Centre of the “Mondino” Institute of Pavia. Diagnosis in the 2 groups was operationally defined according to the ICHD-IIIβ criteria. Patients were evaluated using the Toronto Alexithymia Scale (TAS-20), which uses a five-point Likert response scale and has a three-factor structure consisting of: (1) Difficulty in identifying feelings, (2) Difficulty in describing feeling, and (3) Externally oriented thinking. Demographic and clinical information were collected as well.

## Results

According to multiple binary logistic regression analysis, MIG and MOH patients were comparable in terms of demographic characteristics, whereas they differed for some characteristics of illness (age of migraine onset, duration of illness, frequency of headache), disability and QoL, as well as for depression levels. MOH patients scored higher than MIG on two of the three alexithymia facets, which were those concerning difficulties in identifying (MOH = 19.1±6.7, MIG = 13.8±6.7, p < 0.001) and describing feelings (MOH = 14.4±4.5, MIG = 11.6±4.8, p = 0.003). Groups were instead comparable in terms of externally oriented thinking (MOH = 18.6±4.2, MIG = 18.0±4.1, p = 0.50). Significant correlations resulted between alexithymia and illness characteristics (e.g., headache frequency, perceived disability, and QoL).

## Conclusions

Our results show a specific alexithymic profile in our MOH population. These findings suggest that alexithymia could represent a risk factor in the transformation from episodic migraine into the chronic subtype with medication overuse. Early and appropriate interventions aimed at improving emotional awareness and expression could then represent a further preventive measure to avoid drug-induced headache.

Written informed consent to publish was obtained from the patient(s).

## Conflicts of interests

None.
